# A Textbook of Materia Medica, Therapeutics and Pharmacology

**Published:** 1903-05

**Authors:** 


A Textbook of Materia Medica, Therapeutics and Pharmacology. By George F. Butler, Ph. G., M. D., Professor of Materia Medica and Therapeutics in the College of Physicians and Surgeons, Chicago, Medical Department of the University of Illinois, etc. Fourth edition, thoroughly revised. Octavo volume of 896 pages, illustrated. Philadelphia and London: W. B. Saunders & Co. 1902. [Cloth, $4.00 net; sheep or half-morocco, $5.00 net.]

Butler's work on therapeutics was undertaken with the immediate object of supplying the student of medicine with a clear, concise and practical textbook, adapted for permanent reference no less than for the requirements of the class room. The arrangement of the work was somewhat out of the ordinary —the author believing that the synthetic classification of drugs based upon therapeutic affinities to be the most rational, and one calculated to engage the interest of those to whom the academic study of the subject is wont to offer no little perplexity. On these lines, three editions of Butler have become exhausted and the present—fourth—edition is before the profession. Some changes have been made in this edition—namely, a thorough remodeling of the opening chapters, to bring them into accord with recently discovered biological phenomena.

Naturally, the pharmacology and therapeutics of each drug have been carefully revised, and changes made in the expression of opinion regarding the utility of the newer drugs, notably the synthetics, which larger opinion has modified, resulting in more definite conclusions. The chapters on organotherapy, serum therapy, and cognate branches have been enlarged and thoroughly revised.

Perhaps the most important addition is the chapter devoted to the interesting phases of the newer theories of electrolytic dissociation as revealed by Dresser, Loeb and others. The author believes that it cannot be long before an entirely new analysis of the action of drugs will have to be made. He asserts that pharmacology has acquainted itself fairly well with the superficial attributes of drugs, and the question to be asked is how or why do they act to bring about these results. The chapter is an interesting review of Loeb's work in this department of physical chemistry, and is made clear by the author's inimitable style.

A general index and an extended clinical index serve to make the volume an exceedingly useful one, and it can be heartily recommended to the profession at large. Paper, print and binding are of excellent quality. W. C. K.

